# Angiogenesis as a novel therapeutic strategy for Duchenne muscular dystrophy through decreased ischemia and increased satellite cells

**DOI:** 10.3389/fphys.2014.00050

**Published:** 2014-02-18

**Authors:** Yuko Shimizu-Motohashi, Atsushi Asakura

**Affiliations:** ^1^Stem Cell Institute, University of Minnesota Medical SchoolMinneapolis, MN, USA; ^2^Paul and Sheila Wellstone Muscular Dystrophy Center, University of Minnesota Medical SchoolMinneapolis, MN, USA; ^3^Department of Neurology, University of Minnesota Medical SchoolMinneapolis, MN, USA

**Keywords:** muscular dystrophy, regeneration, angiogenesis, VEGF, Flt-1, satellite cell, *mdx* mice, skeletal muscle

## Abstract

Duchenne muscular dystrophy (DMD) is the most common hereditary muscular dystrophy caused by mutation in *dystrophin*, and there is no curative therapy. Dystrophin is a protein which forms the dystrophin-associated glycoprotein complex (DGC) at the sarcolemma linking the muscle cytoskeleton to the extracellular matrix. When dystrophin is absent, muscle fibers become vulnerable to mechanical stretch. In addition to this, accumulating evidence indicates DMD muscle having vascular abnormalities and that the muscles are under an ischemic condition. More recent studies demonstrate decreased vascular densities and impaired angiogenesis in the muscles of murine model of DMD. Therefore, generation of new vasculature can be considered a potentially effective strategy for DMD therapy. The pro-angiogenic approaches also seem to be pro-myogenic and could induce muscle regeneration capacity through expansion of the satellite cell juxtavascular niche in the mouse model. Here, we will focus on angiogenesis, reviewing the background, vascular endothelial growth factor (VEGF)/VEGF receptor-pathway, effect, and concerns of this strategy in DMD.

## Introduction

Duchenne muscular dystrophy (DMD) is the most common hereditary muscular dystrophy affecting approximately 1 in 5000 live male births (Mendell et al., 2012). It is caused by mutations in *dystrophin* gene located on Xp21 (Monaco et al., 1986), leading to progressive muscle weakness in which respiratory and cardiac failures are the main reasons of their early mortalities. Dystrophin is a protein which forms the dystrophin-associated glycoprotein complex (DGC) at the sarcolemma which links the muscle sarcomeric structure to the extracellular matrix (Davies and Nowak, 2006). When dystrophin is absent due to the gene mutation, muscle fibers become vulnerable to mechanical stretching (Pasternak et al., 1995). Currently there is no curative therapy for this disease and glucocorticoid is the only medication available that slows the decline in muscle strength and function in DMD (Bushby et al., 2010).

Since the identification of *dystrophin* in the mid 1980's (Monaco et al., 1986), several therapeutic approaches have been investigated. Gene replacement with virus vector, induction of protein expression by exon skipping or read through, compensation with dystrophin surrogates, and delivery of muscle stem cells or pluripotent stem cells have been investigated so far (Leung and Wagner, 2013; Rodino-Klapac et al., 2013). Recently, it was announced that phase 3 clinical trial for Drisapersen, an antisense oligonucleotide for exon skipping, could not meet the endpoint of statistically significant improvement (http://www.gsk.com/media.html). Although exon skipping could still be considered as one of the most promising therapeutic approaches available, there is a necessity for developing further therapeutic strategies. We have recently reviewed vasculature-related strategies for DMD, with a major focus on therapeutic methods to increase blood flow in existing blood vessels (Ennen et al., 2013). In the current review, we examine the evidence for reduced formation of blood vessels in DMD muscle and the therapeutic approach to augment angiogenesis by using vascular endothelial growth factor (VEGF)-based strategies. We provide an update of current evidence for changes in vasculature in DMD, approaches available to increase vasculature, and further discuss the pros and cons of the underlying rationale.

## Evidence for vasculature changes in DMD

The necrotic fibers in DMD are often seen in groups, a simultaneous necrosis of contiguous muscle fibers, and it had been thought that this phenomenon was due to local reduction of blood supply by common capillaries in that group of necrotic fibers (Rando, 2001). The dystrophin deficiency in vascular smooth muscle (Miyatake et al., 1989) and absence of nitric oxide synthase (NOS) from the sarcolemma have indicated that DMD muscle is subjected to impaired blood flow (Brenman et al., 1995; Rando, 2001; Ennen et al., 2013).

More recent studies demonstrate that *mdx* mice muscle has decreased vascular density. Immunostaining of arterioles has revealed decreased vascular density in heart and gracilis muscles of *mdx* mice (Loufrani et al., 2004). Matsakas et al., have visually shown that vasculature of the tibialis anterior (TA) in *mdx* mice is reduced compared to wild type by microfil-perfused whole mount imaging (Matsakas et al., 2013).

Another report shows that angiogenesis is impaired in *mdx* mice, not only in muscle, but systemically, which was proven by laser Doppler perfusion imaging in the hind limb ischemic model, VEGF induced neovascularization quantification in the corneal model, Matrigel subcutaneous angiogenic assay, and quantification of tumor growth and vascularization in the tumor implant model (Palladino et al., 2013).

Rhoads et al., have indicated satellite cells isolated from aging and *mdx* mice exhibit decreased expression of hypoxia-inducible factor 1α (HIF-1α) and VEGF and reduced capacity to promote angiogenesis *in vitro*, using a co-culture model of conditioned media from *mdx* mice or wild type satellite cells co-cultured with microvascular fragments (MVFs) (Rhoads et al., 2009, 2013). They also demonstrated that VEGF mRNA expression was decreased in proliferating satellite cells in dystrophic muscle. Furthermore, hypoxic conditions increase VEGF mRNA expression in satellite cells (Flann et al., 2013).

Taken together, there is a rationale to believe that DMD has significant defect in vasculature in terms of its quality, quantity, and angiogenesis, and that the muscles are under an ischemic condition.

It should be critically discussed whether the vascular change seen in DMD is a primary effect of the disease or not. Studies of blood perfusion in *mdx* mice indicate age or disease progression may have a major effect in vascular changes. A study with 2-month-old *mdx* mice showed increased blood flow compared to the wild type control in the hindlimb ischemia model, whereas older mice of 6 months showed decreased blood flow (Straino et al., 2004; Palladino et al., 2013). In wild type mice, it has been indicated that aged mice have a reduced response to angiogenesis after ischemia (Palladino et al., 2011, 2012). These data imply that the vascular changes seen in *mdx* mice could be the physiological response to aging and disease progression, rather than being the primary disease effect.

In one study of gene expression profiling of DMD patients' muscles, VEGF appears to be lower in DMD than the controls (Bakay et al., 2002), whereas in another study, a significant difference was not shown (Haslett et al., 2002). This inconsistency may be due to the data analysis and differences in experimental design (Haslett et al., 2002). It also has been reported that blood VEGF levels are significantly lower in DMD with mean age of 8.1 ± 1.9 years (Abdel-Salam et al., 2009). Others reported that VEGF levels in serum samples from DMD patients with mean age of 14.6 ± 0.8 years was elevated (Saito et al., 2009). Although the latter study was not compared to age-matched controls, these data imply that VEGF secretion fluctuates according to age or disease progression.

Further studies are required to elucidate the mechanism of vascular change in DMD, however, the studies designed to improve vasorelaxation capacity (Ennen et al., 2013) or to increase vasculature in order to improve tissue perfusion in DMD animal models have demonstrated the amelioration of dystrophic phenotypes (Asai et al., 2007; Verma et al., 2010; Kawahara et al., 2011). As far as we are aware, the approaches to increase vascular density have not been applied to humans yet, with animal model studies showing promising results for DMD therapy (Table 1).

**Table 1:** **Different approaches that could increase vascular density in DMD model animals**.

**Approach**	**Age of mice at treatment onset**	**Outcome**	**References**
VEGF overexpression via AAV gene transfer in *mdx* mice	4 weeks	Increased capillary density in regenerating areas	Messina et al., 2007
		Reduced necrotic fiber areas	
		Increased regenerative fiber areas	
		Increased forelimb strength	
VEGF overexpression via muscle-derived stem cell (MDSC) transplantation into *mdx/scid* mice	8–10 weeks	Increase in angiogenesis	Deasy et al., 2009
		Increase in muscle regeneration	
		Reduction in fibrosis	
Genetic modulation of VEGF receptor (Flt-1) level in *mdx* and *mdx/utrn^−/−^* mice	2–3 months	Increased vascular density	Verma et al., 2010
		Decreased muscle membrane permeability	
		Less area of fibrosis and calcification	
		Decreased centrally located nuclei	
		Increased tissue perfusion	
		Improved maximum isometric force and whole-body tension analysis	
Overexpression of estrogen-related receptor-γ (ERRγ)	6–8 weeks	Enhanced vasculature and blood flow	Matsakas et al., 2013
		Increased number of oxidative myofibers	
		Improved exercise tolerance	
Mesoangioblast transplantation into the heart of *mdx/utrn^−/−^* mice	4–6 weeks	Prevented onset of cardiomyopathy	Chun et al., 2013
		Increased capillary in the heart	
Treatment with aspirin	4 weeks (treatment continued for 7 months)	Increased vascular density	Palladino et al., 2013
		Decreased muscle membrane permeability	
		Less area of fibrosis	
		Increased numbers of regenerating fibers	
		Increased tissue perfusion	
		Improved resistance to physical exercise	

## Different approaches to increase vascular density in DMD

### VEGF overexpression

VEGF-A (also known as VEGF) is crucial for blood vessel formation during early embryogenesis (Shibuya, 2013). It binds to VEGFR-1 (Flt-1) and VEGFR-2 (Flk-1) which are the membrane-spanning tyrosine kinase receptors and both have pro-angiogenic effects. The major pro-angiogenic effect is generated through Flk-1, on the other hand, Flt-1 can act negatively in angiogenesis. Although there is higher affinity to VEGF in Flt-1 compared to Flk-1 (Sawano et al., 1996), its kinase activity is lower than Flk-1. Besides full length Flt-1, there is a truncated soluble type (Kendall and Thomas, 1993) which lacks transmembrane and tyrosine kinase domains, and is considered to act as decoy receptor of VEGF.

Overexpression of VEGF via AAV gene transfer in muscles of *mdx* mice showed an increased number of capillaries/fibers (Messina et al., 2007). The treated *mdx* mice had increased forelimb strength, reduced necrotic fiber areas, and increased regenerative fiber areas. Increased capillary density was only seen in regenerating areas. Although this study also highlights the role of direct pro-regenerative effect of VEGF to skeletal muscle, they discuss the possible beneficial effects of VEGF-induced muscle neovascularization on dystrophic muscle as (1) promoting macrophage recruitment and removal of cellular debris; (2) increasing release and circulation of factors secreted by mononuclear cells and activating myogenic cells, and (3) increasing the recruitment of bone marrow derived mononuclear cells, which in turn release factors that activate the myogenic process.

### VEGF receptor modulation

**Figure 1:**
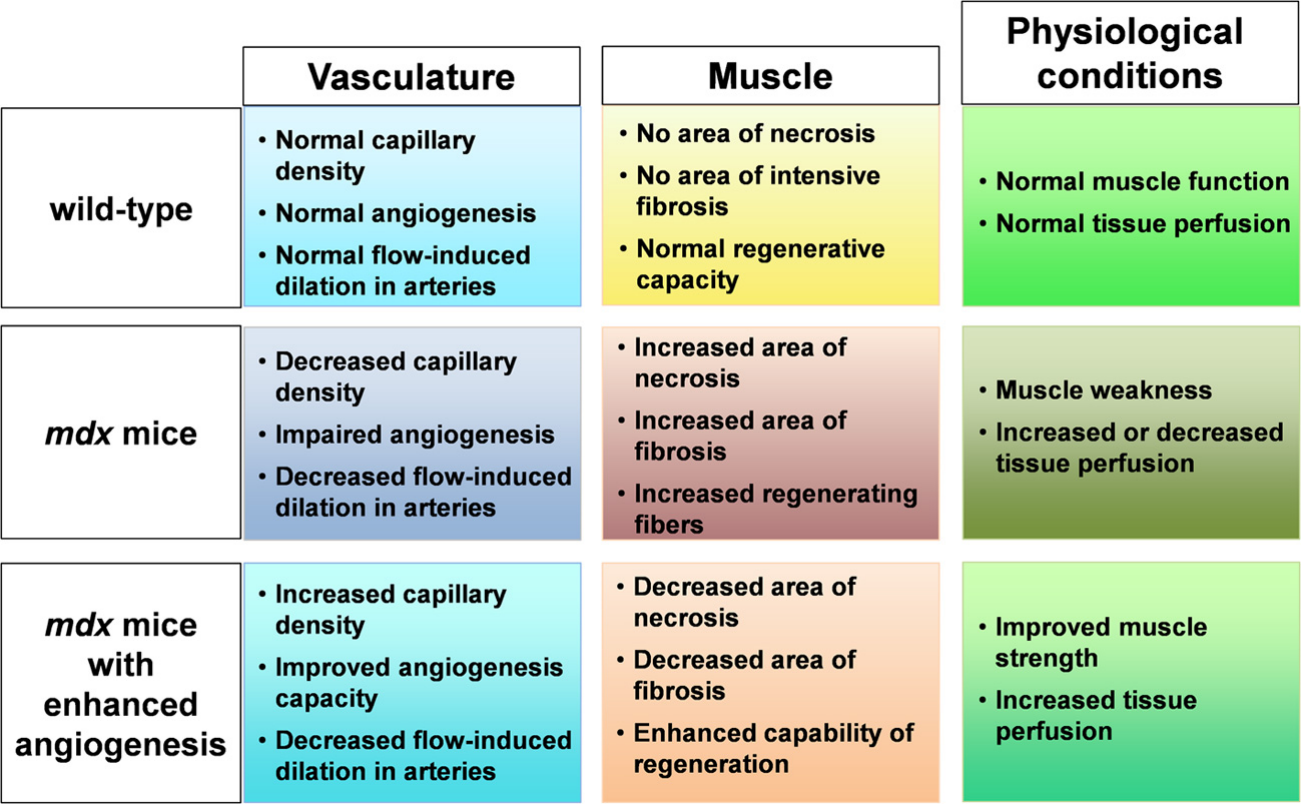
**Schematic figure of relationship between muscle, capillary, and physiological condition**. In *mdx* mice, capillary density is decreased, angiogenesis is impaired with decreased flow induced dilation in arteries. When angiogenesis is enhanced in *mdx* mice, increased capillary density and improved angiogenesis capacity leads to decreased area of necrosis and fibrosis and increased regenerative capacity in muscle accompanied with increased tissue perfusion and improved muscle weakness.

We recently reported that *mdx* mice crossed with heterozygous *Flt-1* gene knockout mice (*Flt-1*^+/−^) showed increased vascular density and ameliorated phenotype compared to control *mdx*:*Flt-1*^+/+^ mice (Verma et al., 2010; Ennen et al., 2013). Our data showed that when *Flt-1*^+/−^ mice were compared with their wild-type (*Flt-1^+/+^*) littermates, *Flt-1*^+/−^ mice had a significantly increased number of endothelial cells (ECs) and increased tissue perfusion in TA muscle. When *Flt-1*^+/−^ mice were crossed with *mdx* mice to create double mutant *mdx* mice with the heterozygous allele for *Flt-1*^+/−^ (*mdx:Flt-1*^+/−^) and compared with their littermates *mdx* mice with control Flt-1 (*mdx:Flt-1^+/+^*) mice, *mdx:Flt-1*^+/−^ mice exhibited a higher number of ECs, increased blood flow and improved muscle function. In these double mutant mice (*mdx:Flt-1*^+/−^), muscle histology suggested decreased fiber turnover and increased fiber stability. Importantly, *mdx:Flt-1*^+/−^ mice display increased number of satellite cells in the muscle compared to *mdx:Flt-1^+/+^* mice. Satellite cells are a muscle stem cell population in adult skeletal muscle and are essential for postnatal muscle growth and regeneration. As muscle ages or is afflicted by disease, muscle regeneration is impaired due to the decreased number and decreased differentiation capacity of satellite cells (Mounier et al., 2011). Therefore, it is possible that an increase in the vascular niche might promote muscle regeneration via stimulation of satellite cell proliferation or survival. These data strongly suggest that *Flt-1* haploinsufficiency ameliorates muscular dystrophy phenotype by developmentally increased vasculature in *mdx* mice, further implying the possibility of VEGF receptor modulation as a therapeutic strategy (Figure 1).

### Other approaches that can increase vascular density in muscle

Estrogen-related receptor-γ (ERRγ) is known to be highly expressed in skeletal muscles, and it has been demonstrated that ERRγ can induce angiogenic factors including VEGF to increase angiogenesis in muscle (Narkar et al., 2011). Matsakas et al., reported that ERRγ expression and downstream metabolic and angiogenic target genes are down-regulated in the skeletal muscles of *mdx* mice (Matsakas et al., 2013). In this study, overexpression of ERRγ selectively in the *mdx* mice skeletal muscle could enhance vasculature, blood flow, and oxidative myofibers, and improve exercise tolerance.

Palladino et al., hypothesized that aspirin has beneficial effects on the angiogenic properties of ECs in dystrophic mice, due to its ability to enhance NO release from vascular ECs and protective effect on ECs via the NO cGMP pathway (Palladino et al., 2013). Treatment with aspirin could enhance production of NO and cGMP, and long-term low dose aspirin could increase capillary density, improve resistance to physical exercise, and muscle fiber permeability.

It is known that exercise training promotes many adaptations in skeletal muscle, including enhanced angiogenesis (Andersen and Henriksson, 1977; Gavin et al., 2004). Although excessive exercise may exacerbate the DMD phenotype, these studies imply that an adequate amount of exercise may be beneficial to DMD.

### Concerns regarding VEGF administration and its receptor modulation

Upon targeting the VEGF/VEGF receptor pathway for therapy, the greatest concern would be whether the newly generated vasculature was morphologically and functionally sound *in vivo*. Although VEGF is a well-known factor for angiogenesis, it was first described as vascular permeability factor, and these vascular permeability-producing effects of VEGF may have profound negative consequences in ischemic disease by augmenting the level of infarcted tissue (Senger et al., 1983; Weis and Cheresh, 2005). An *in vivo* imaging study performed in rat skeletal muscle with prolonged expression of VEGF via AAV vector revealed impaired perfusion of the tissue (Zacchigna et al., 2007). Despite the histological evidence of neoangiogenesis, resting muscle blood flow measured by positron emission tomography did not improve, and moreover, post-exercise muscle blood flow measurements showed decreased perfusion. This phenomenon was explained by the formation of leaky vascular lacunae which accounted for the occurrence of arteriovenous shunts that excluded the downstream microcirculation.

Although the precise role of *Flt-1* in postnatal and adult tissue angiogenesis still remains ambiguous, a recent study demonstrated Cre-loxP-mediated conditional knockout mice with ablated *Flt-1* expression in neonatal and adult periods displaying increased angiogenesis in various tissues including cornea, lung, heart, brain, kidney, and liver (Ho et al., 2012). Moreover, the vasculature seen in the conditional *Flt-1* knockout mice matured and perfused properly. These findings are especially encouraging for developing therapeutic strategies targeting VEGF/Flt-1 interaction.

### Combination therapy of delivering an angiogenesis and myogenesis factor

Borselli et al. reported that a combination of VEGF to promote angiogenesis and insulin-like growth factor-1 to directly promote muscle regeneration could induce functional recovery in an ischemic injured skeletal muscle in a more prominent manner than VEGF alone (Borselli et al., 2010). It is also noteworthy that in their study, sustained delivery of the factors via injectable gel was effective, whereas bolus delivery did not show any benefit in terms of angiogenesis, regeneration, and muscle perfusion. These data imply alternative strategies may be used to obtain more benefits of angiogenesis therapy in DMD.

## The significance of VEGF/VEGFR pathway in DMD

### Direct effect of VEGF on skeletal muscle

Other than the pro-angiogenic effect, a pro-myogenic effect of VEGF has been reported in skeletal muscle; however, the effect of VEGF on muscle cell function is still unclear. The presence of VEGF receptors has been investigated by several groups. *In vitro* experiments using C2C12 cells and primary mouse myoblasts showed that both Flt-1 and Flk-1 were detected in western blotting analysis and RT-PCR (Germani et al., 2003; Arsic et al., 2004), and their expression levels were modulated during the course of differentiation. With normal muscle tissue, immunohistochemistry data indicated Flt-1 and Flk-1 expression levels in muscle fibers are either very low or null (Arsic et al., 2004; Wagatsuma et al., 2006; Messina et al., 2007), but once the muscle was subjected to experimental injury, both receptors became detectable in satellite cells and regenerating muscle fibers (Arsic et al., 2004; Wagatsuma et al., 2006).

When VEGF was administered to C2C12 cells, cell migration and survival were enhanced (Germani et al., 2003). Others have shown an increased number of myotubes expressing myosin heavy chain (MHC), a myogenic differentiation marker, in differentiated C2C12 cell cultures supplemented with VEGF (Arsic et al., 2004). These MHC-positive fibers contained more multinucleated myofibers while non-VEGF-treated cells consisted mainly of mononuclear myocytes. In addition to these findings, VEGF-treated cells had significantly longer mono- or multinucleated MHC-positive cells. Also, when C2C12 cells were treated with an apoptosis-triggering agent, VEGF could decrease the fraction of cells expressing markers of necrosis. It also has been reported that when human myogenic precursor cells were incubated with VEGF, there was an increase in cell density (Christov et al., 2007).

Taken together, it is indicated that VEGF may have a direct proliferative effect on muscle in addition to promotion of myogenic fiber growth and an anti-apoptotic effect.

### The pro-angiogenic and pro-myogenic effects of the VEGF/VEGFR pathway play an important role in the regenerative process in muscle

Recent studies highlight the importance of angiogenic and regenerative effects of VEGF in muscle regeneration (Beckman et al., 2013; Bobadilla et al., 2013). Mechanical stimulation (MS) can increase VEGF secretion (Payne et al., 2007; Cassino et al., 2012), and MS increases the effectiveness of tissue repair in muscle-derived stem cell (MDSC) transplantation experiments in *mdx/scid* mice (Beckman et al., 2013). Inhibition of VEGF with soluble Flt-1 (sFlt-1) or short hairpin RNA (shRNA) in mechanically stimulated MDSCs (MS-MDSCs) resulted in reduction of cells' differentiation and angiogenic capacity within the transplantation area, both of which are increased in MS-MDSC transplantation cases (Beckman et al., 2013).

Bobadilla et al., indicated metalloproteinase-10 (MMP-10) has a role in muscle regeneration in injured or dystrophic muscle through VEGF/Akt signaling (Bobadilla et al., 2013). MMP-10 protein levels were upregulated in *mdx* mice, and ablation of MMP-10 in *mdx* mice deteriorated the dystrophic phenotype. Although it was not significant, MMP-10 knockout mice (MMP-10 KO) crossed with *mdx* mice had fewer arterioles in the muscle, and MMP-10 KO had significantly lower number of arterioles than the wild type control. Various MMPs induce VEGF secretion, and in their study, MMP-10 mRNA silencing in injured wild type muscle, a decrease in VEGF protein level was observed, while treatment with recombinant human MMP-10 showed elevated VEGF (Mott and Werb, 2004; Bobadilla et al., 2013). Collectively, these data suggest that pro-myogenic and pro-angiogenic effects of VEGF/VEGFR play an important role in the muscle regeneration process in DMD.

## Endothelial cell and satellite cell relationship

ECs and factors secreted by them are able to induce satellite cell proliferation and survival. Christov et al., reported that in adult normal muscle, satellite cells are located preferentially close to capillaries, and reciprocally interact with ECs to support the angio-myogenesis relationship (Christov et al., 2007). Their study further indicated that there is a correlation between capillary and satellite cell numbers. In muscle specimens from patients with amyopathic dermatomyositis (aDM), an inflammatory disease, capillary density in the muscle is decreased but is spared from both myofiber damage and inflammation. Interestingly, a proportionate decrease in the mean satellite cell number per myofiber was also observed. Conversely, athletes' muscles, which have workload-induced increased capillary density, showed increased numbers of both capillaries and satellite cells per myofiber. Indirect coculture using chambers with human umbilical vascular ECs (HUVECS) or human microvascular ECs (HMECS) cultured in the upper chamber, and separated with porous filter, human myogenic precursor cells cultured in the lower chamber, revealed that an endothelial cell monolayer could increase myogenic cell growth through soluble factors. EC derived IGF-1, HGF, bFGF, PDGF-BB, and VEGF were indicated as effectors for myogenic cell growth promotion. These data suggest that satellite cell and muscle regeneration are under influence of vasculature and that they receive supportive cues from ECs to expand and further commit and differentiate (Mounier et al., 2011).

## Vascular network in DMD cardiac muscle and possible effect of angiogenesis

In *mdx* mice, it has been reported that there is reduced vasculature in cardiac tissue compared to the wild-type (Loufrani et al., 2004). A recent study of postnatal *Flt-1* gene ablation, in combination with left anterior descending artery ligation to create ischemic cardiomyopathy, showed reduced infarction size and increased capillary density after ligation (Ho et al., 2012). Chun et al., could prevent the onset of cardiomyopathy by transplanting mesoangioblast stem cells into the heart of *mdx/utrn^−/−^* mice. They reported increased number of capillaries in the treated hearts, indicating a possible positive effect on angiogenesis, a well-known indirect effect of stem cell therapy (Chun et al., 2013). These data imply that angiogenesis may be beneficial also for DMD cardiomyopathy.

## Conclusions

Mounting evidence for DMD vascular abnormality indicates that vascular therapy is a logical approach. Currently, the most frequently used method to increase vasculature is by modulating VEGF/VEGFR pathways. DMD muscle may benefit from angiogenesis in multidimensional aspects. Increasing capillary density simply resolves ischemia. Direct regenerative and anti-apoptotic effects of VEGF can be expected. Increased vascular niches that house satellite cells imply enhanced proliferation of satellite cells under the influence of supportive cues from vasculature.

The concern would be whether therapeutically generated vasculature is morphologically and functionally beneficial. Although this issue must be investigated before treating human DMD, increasing data currently provide strong support that angiogenesis is a promising therapeutic strategy for DMD.

### Conflict of interest statement

The authors declare that the research was conducted in the absence of any commercial or financial relationships that could be construed as a potential conflict of interest.
